# Mapping intersectionality in dementia care research: a decadal scoping review

**DOI:** 10.3389/frdem.2026.1779670

**Published:** 2026-09-03

**Authors:** Martina Roes, Franziska Laporte Uribe, Simone Anna Felding, Georgina Charlesworth, Nicole Müller, Saloua Berdai, Jem Bhatt, Kuebra Altinok, Viktoria Peters-Nehrenheim

**Affiliations:** 1 German Center for Neurodegenerative Diseases (DZNE e.V.), Witten, Germany; 2 Faculty of Health, Department of Nursing Science, University of Witten/Herdecke, Witten, Germany; 3 Department of Public Health, University of Copenhagen, Copenhagen, Denmark; 4 University College of London (UCL), London, United Kingdom; 5 University College Cork (UCC), Cork, Ireland; 6 Society and Ageing Research Lab, Vrije Universiteit Brussels, Brussels, Belgium; 7 Faculty for Health Care, University of Bielefeld, Bielefeld, Germany

**Keywords:** dementia, intersectional analysis, intersectionality frameworks, methodological analysis, research gaps

## Abstract

**Background:**

In the past decade, an increasing number of studies have addressed health care disparities and the complex determinants of care for diverse populations. At the same time, it remains unclear how social categories (e.g. gender, ethnicity, migration status, socioeconomic position) intersect with health care disparities for people living with dementia and may lead to experiences of inequity and inequality.

**Aim:**

This scoping review analyzes the use of intersectionality frameworks and intersection analyses in dementia care research over the past decade (2015–2025).

**Method:**

We followed Arksey and O'Malley's six-stage scoping review methodology, extended with PRISMA guidelines and used the McCall framework to critically synthesize the included articles.

**Results:**

We identified 14 articles that met our criteria for applying an intersectionality framework to analyze data. The most mentioned intersectionality frameworks in publications are from Crenshaw (*n* = 8), McCall (*n* = 7) as well as Hankivsky (*n* = 3) and Collins (*n* = 3). The target groups varied; mostly, the focus was on migrants, ethnicity, or sex/gender.

**Conclusion:**

Our results indicate a shift in dementia care research toward recognizing lived experiences shaped by multiple axes of identity and contexts. At the same time the primary analytical focus lies in the intersection of two axes of identity (such as sex/gender and ethnicity/migration). A detailed analysis of power dynamics within the healthcare system or the society, and how they may contribute to the experiences of the target group, was mostly not conducted or only mentioned that there is a need to do so.

## Introduction

Intersectionality, a concept first developed by Crenshaw (1989) highlights, how different systems of power intersect and interact with axes of identity and therefore lead to specific disadvantages (e.g., low income) or advantages (e.g., wealthy people) for population groups or individuals. She criticizes approaches that analyze social problems through only one lens (such as ethnicity), arguing that the complexity of real life needs to be considered in order to understand people's experiences. For example, the experiences of Black women cannot be understood by only applying the concept of racism. Rather, analyses need to consider how experiences are shaped by the conjunction of ethnicity and other social constructs and their respective layers of discrimination (Carbado et al., 2013). Crenshaw's framework emphasizes that the intersecting dynamics of the axes of identity and societal norms, laws and/or policies must be analyzed together to be able to identify and understand experiences of inequality and inequity.

Crenshaw (1989) characterized intersectionality as an analytical tool and a mindset about power within our societies, illustrated by the positioning of Black women (Crenshaw, 1991) and distinguishes between structural and political intersectionality. Following the publication of her work, the concept of intersectionality has been theorized in different ways: McCall (2005) focuses on a conceptional approach and introduces anti-, inter-, and intra-categorial approaches to study inequalities. Her framework can be used to analyze intersectional power dynamics. Hankivsky (2014) adopts an equity-policy perspective and notes that power relations and operational structures can be analyzed across micro-, meso and macro levels. Her framework, Intersectionality-Based Policy Analysis (IBPA), can be applied to analyze research, policy, and practice. Collins (2019) defines intersectionality as a critical social theory with core constructs (e.g., relationality, complexity). Her framework provides a formalized theoretical architecture of intersectionality across contexts (Collins et al., 2021). Hancock (2016) views an intersectionality framework as a research paradigm, with a specific focus on political science and policy research. She points out that it is necessary to move from *what intersectionality is* toward *how intersectionality can reconfigure research* (Hancock, 2007). Bowleg (2012) links intersectionality to public health and highlights how interlocking systems of power and oppression shape the lived experience of individuals. Her focus lies in institutional critique and the need for policy-level-analysis (Bowleg, 2017). These intersectional frameworks emphasize systems of power that intersect with constitutive categories and reject decontextualized models of inequality and inequity.

The experiences of people living with dementia (PLWD) are heterogeneous and differ at the intersection of the axes of identity (e.g., age, ethnicity), social geography (e.g., where one lives) and societal context (e.g., income). Analyzing complex individual experiences through the lens of intersectionality enables researchers to uncover inequalities/inequities and their dynamics that would otherwise remain invisible within the health care system. Therefore, it is important to understand dementia not only from the perspective of a neurodegenerative disease, but as a social construct, arguing that dementia is a label that shapes how people living with dementia are treated by society. Experiences, such as stigma, taking away autonomy and citizen rights, ignoring personhood and decision-making capacity, suggest that dementia is also socially constructed (Parker et al., 2021). Applying an intersectional lens in dementia care research can support improvements to health care systems to make them more responsive to populations with overlapping vulnerabilities, to move beyond simplistic group comparisons, and instead to ask how power, marginalization, and privilege are produced in everyday care and how the resulting disadvantage can be addressed structurally.

Intersectionality began to explicitly enter dementia care research around the early 2010s, although references to preliminary ideas of “multiple disadvantage” or “cultural diversity” appeared earlier. However, systematic engagement with intersectionality frameworks did not become prominent in the literature until the mid-2010s (Hengelaar et al., 2023). Early dementia care studies applying an intersectional lens addressed topics such as gender and citizenship (Bartlett et al., 2018), ethnicity or migration status (Dilworth-Anderson and Anderson, 1994) and/or language skills (Koehn et al., 2016), which impact access to the healthcare system or to a timely diagnosis. These early studies analyzed differences in care experiences within different health care systems (Hulko, 2004), but mostly considered aspects of identity in an additive manner (Hicks et al., 2025; O'Connor et al., 2010).

The growing use of intersectionality as an analytical framework in dementia care research aims to identify and address gaps in perspectives, exclusion, and discrimination faced by marginalized groups. Therefore, the aim of our scoping review is to provide an overview of the use of intersectionality frameworks in dementia care research over the past decade (2015–2025).

## Methods

Our review follows (Arksey and O'malley, 2005) six-stage scoping review methodology, extended with the PRISMA guidelines. Stage 1: identifying the research question; Stage 2: identifying relevant studies; Stage 3: selecting studies; Stage 4: charting the data; Stage 5: collating, summarizing, and reporting the results; and Stage 6: consulting with experts.

### Stage 1 research question

Our guiding research question was the following: which intersectionality frameworks have been applied in dementia care research published between 2015 and 2025?

### Stage 2 search strategy

Searches for relevant articles were conducted in six different databases (PubMed, PsychInfo, Scopus, CINAHL, Embase and Web of Science). The search string was adjusted for the different data bases (see Supplement 1) with overarching terms being: (“dementia” OR “Alzheimer^*^”) AND (“intersectionality” OR “intersectional” OR “multidimensional identity” OR “structural inequality” OR “disparities” OR “migrants” OR “minority group” OR “social determinants”) AND (“care” OR “caregivers” OR “healthcare” OR “support” OR “service”) AND (“framework” OR “analysis” OR “approach” OR “model”). Articles were retrieved, and duplicates were deleted. The articles were downloaded into an EndNote file.

### Stage 3 study selection

We followed the Population, Concept, and Context (PCC) framework (Peters et al., 2022) (Table 1) in which the *population* included people living with dementia or family/informal carers of people living with dementia or health professionals taking care of people living with dementia. Articles must contain reference to an intersectional framework (*concept*), applied to a dementia care setting (*context*). Papers were eligible if they were original papers or reviews (peer reviewed) with a clear link to dementia care (research), published between 2015–2025 in either English or German. Gray literature, conceptual papers, editorials, abstracts or posters and dissertations were excluded, as were biomedical articles.

**Table 1:** PCC criteria.

PCC	Inclusion	Exclusion
Population	People with dementia or family/informal carers of people with dementia or health professionals taking care of people with dementia	Articles/studies that do not focus on people with dementia
Concept	The phenomenon of interest is the application of an intersectional framework in dementia care research	Articles/studies not including an intersectional framework in dementia care research
Context	Dementia care research contexts	No dementia care research setting

## PCC criteria

Since the number of articles identified for title/abstract (TiAb) screening was relatively low, we used only the EndNote file and reviewed the identified articles independently (MR and FLU). In case of conflict a third researcher (VPN) was included to reach consensus. Reference lists of excluded reviews were searched, with potentially eligible studies screened.

### Stage 4 data charting

MR extracted data from all the articles and discussed articles with the co-authors. Data extraction focused on authors, year, title, applied intersectionality framework, research question, target group, key outcomes, and author conclusions.

### Stage 5 collating and summarizing

All included articles were analyzed by searching for any mention and application of an intersectionality framework. These questions were used to guide the analysis:

- Aim/research question: does the aim or research question mention an intersectionality framework?
- Sample size: is the target group clearly described? Which characteristics are described?
- Methods: which methods were used to operationalize the chosen intersectionality framework?
- Axes of identity: which identity markers were been considered/analyzed? Which contextual details are been provided?
- Power dynamics: are power relations mentioned/described between axes of identity and the social system / the health care system? Are experiences of inequity or inequality mentioned / described?

### Stage 6

Consultation was conducted with the members of the INTERDEM taskforce “Context, Culture, and Intersectionality” during our scheduled meetings. INTERDEM is a European Dementia Care Research Network (https://interdem.org), founded in 1999. The network consists of different taskforces. The authors of this scoping review are all members of the taskforce “Context, Culture, and Intersectionality,” which was initiated in 2019 (https://interdem.org/?page_id=7329).

## Results

**Figure 1:**
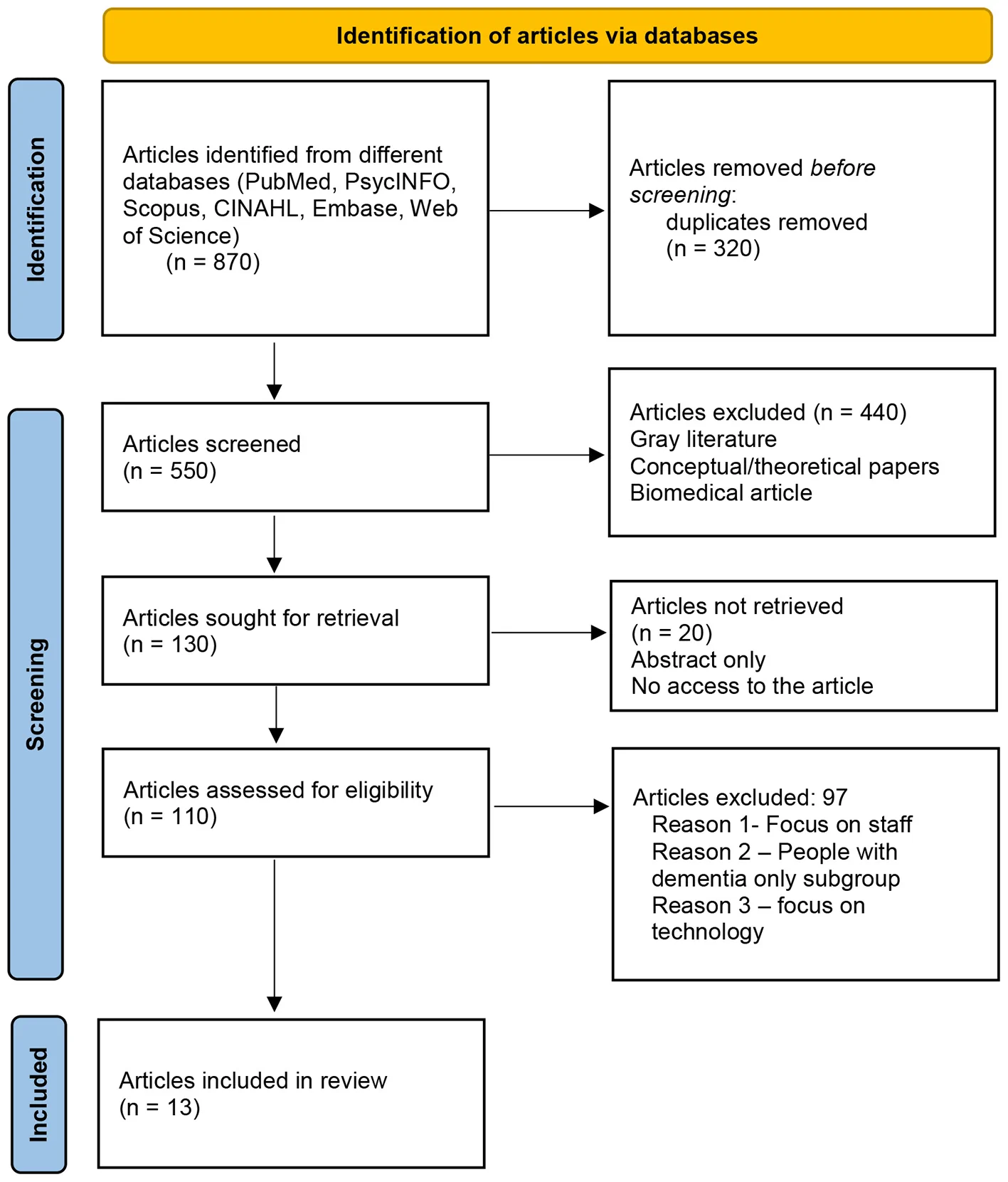
PRISMA flowchart.

The results are presented in three parts: (1) study identification, including a PRISMA flowchart (Figure 1); (2) the data extraction table showing the study characteristics of included articles; and (3) a critical reflection on the respectively applied intersectionality frameworks.

## PRISMA flowchart

### Study identification

In total 870 articles were retrieved. Among these, 320 were duplicates, 550 articles were subjected to Ti/Ab screening by MR, and the first 10 articles were discussed with a 2^nd^ researcher (FLU or VPN). 110 articles were eligible for full text screening. Thirteen articles were included in this review (Table 2 and 3).

**Table 2:** Study characteristics (incl. intersectionality framework) (alphabetical order).

Authors	Title of the article	Intersectionality Framework	Research Design & methods	Population	Key Findings
Ahmad et al. (2022)	Dementia care-sharing and migration: An intersectional analysis of family care practices	Crenshaw (1989, 1991) [original intersectionality concept] and Prins (2006) [a systemic approach foregrounding structure and a constructionist approach foregrounding agency] and Phoenix (2011, 2006) [that researchers should inductively decide which intersections matter in context]	**Qualitative**: Life-story interviews and shadowing	Migrant family carers providing care to relatives with dementia	•Highlights unequal care burdens for migrant women and how structural conditions (labor markets, welfare rules) shape intra-familiar negotiations •how gender, migration trajectories, class and legal status interact to distribute and constrain care roles
Altinok et al. (2025)	Intersectional research on dementia care for post-migrants and ethnic minority groups: a scoping review	Crenshaw (1989, 1991) [original intersectionality concept]	Scoping **review**	Post-migrants & ethnic minorities people with dementia and carers	•Preferences are not linked to intersectional determinants •Personal preferences are shaped by intersecting cultural values, language barriers, immigration processes, shifting socioeconomic positions and gender roles •Underscores system-level neglect of these intersections in service planning.
Ghanem et al. (2025)	Key considerations for applying intersectionality theory to partner and stakeholder engagement in public health	Hankivsky (2012) [Intersectionality-Based Policy Analysis (IBPA) Framework] to provide a method on how to analyse health policy (reports)	Literature **review**	Different stakeholders	•Calls for incorporating intersectionality principles in partner-stakeholder engagement (PSE) in public health •Encouraging critical reflection on the who, why, and how of PSE
Mwale et al. (2025)	Privileges, and permissions: theorising intersectionality and cultures of control in the care of people living with dementia in acute hospital settings	Phoenix (2006) concept of intersectionality and relational power	**Ethnographic** observations	Dementia patients in acute hospitals in UK	•Institutional power systems drive inequalities and intersectionality exposes structures •Critique and deconstruct frameworks of exclusion and control embedded in hospital cultures
Liu et al. (2022)	Caregiving Burden Among Caregivers Through Lens of Intersectionality	Crenshaw (1989) [original intersectionality concept] and Hankivsky (2012) [health equity intersectionality framework] an McCall (2005) [methodology]	**Quantitative**: Logistic regression	Informal caregivers	•Ethnicity/gender intersections yield distinct burdens •Tailored interventions are needed
Roes et al. (2022)	Intersectionality and its relevance for research in dementia care of people with a migration background	Bowleg (2012), Collins (2019) [intersectionality concept]; Crenshaw (1989) [original intersectionality concept]; Hankivsky (2012) [Intersectionality-Based Policy] Analysis (IBPA); McCall (2005) three different categorical approaches	Perspective Paper	People with dementia and migration history	•Interlocking identities reveal systemic care barriers •Advancing intersectionality is needed to understand exclusion from dementia care
Sherman et al. (2023)	Intersectionality in nursing research: A scoping review	Provides a brief overview of different intersectionality frameworks (e.g. **Crenshaw, Bowleg**), not dementia research specific	Scoping **review**	Nursing/dementia care recipients	•More explicit use of intersectionality frameworks needed
Wong et al. (2024)	Living with dementia: Exploring the intersections of culture, race, and dementia, stigma	Crenshaw (1989) [original intersectionality concept] and Collins (2019) [intersectionality as a critical social theory] and Applying McCall's (2005) intra-categorical approach	**Qualitative**: interviews	Immigrant PWLD and caregivers living in Canada	•Identifies how racism, cultural norms, immigration histories and cognitive impairment interact •Producing layered stigma, social exclusion, and barriers to support •Highlights power in health encounters and service access.

Bold values highlighting the authors.

**Table 3:** Study characteristics, no specific intersectionality framework (alphabetical order).

Authors	Title of the article	Focus on intersections, not explicit an intersectional framework	Research Design & methods	Population	Key Findings
Bartlett (2022)	Inclusive (social) citizenship and persons with dementia	**Single-axis2** gender lens to argue for includes social citizenship	**Qualitative**: Go-along walking followed by sitdown interviews	People with dementia and caregivers in England	Identity and agency reconstructed across intersecting axes • Agency depends on intersectional •recognition
Bartlett et al. (2018)	Gender, citizenship and dementia care: a scoping review of studies to inform policy and future research	Single-axis gender lens (e.g. Lister (2003); **Lehter by 2003**) to expose gendered norms, policy discourses and care practices	Scoping **review**	Family carers of people with dementia	• Need to address gender, migration, policy together •• Intersectionality essential for inclusion
Misiura et al. (2023)	Intersectionality in Alzheimer's Disease: the role of female sex and Black American race in the development and prevalence of AD	**intersecting axes** of sex and race, such as female biological sex, Black American race/ethno-racial identity, **and a set of social determinants of health** (poverty, education, discrimination, allostatic load, vascular risk factors, access to care, under-representation in trials)	Selective literature **review**	Black female Americans with Alzheimer's Disease	• Social and biological factors, such as allostatic load and hormone use, intersect to increase Alzheimer's •Disease risk
Shippee et al. (2024)	Scoping literature review: experiences of sexual and gender minority older adults with diagnoses of dementia, who use residential longterm services and supports	focus on **how** individual, interpersonal, organizational, community and policy-level **factors interact** to shape health outcomes (e.g. McLeroy et al., 1988)	Scoping **review**	Sexual & gender minority older adults with dementia	• Sexual, gender, minority (SGM) older adults encounter compounded discrimination • Risk is magnified (e.g., transgender, racial minorities, HIV+) • Facility and staff often remain •inadequate
Zacher et al. (2023)	Geographic Patterns of Dementia in the United States: Variation by Place of Residence, Place of Birth, and Subpopulation	**Intersection of place and subpopulation** using a life course and social special health disparity lens (e.g. Akushevich et al., 2021)	**Quantitative**: Logistic regression	People with dementia living in the US	• Disadvantage and nuanced patterns of dementia prevalence embedded within •place and subpopulation groupings

Bold values highlighting the intersectional aspect.

#### Study characteristics (alphabetical order)

##### The applied intersectionality frameworks

In the 13 articles included in this scoping review, several intersectionality frameworks were applied. To obtain a better understanding of the frameworks, brief explanations are provided below: axes of identity and social positions, analysis of power dynamics and understanding of inequity and inequality. To identify *axes of identity and social positions*, we defined relevant social categories (axes) and linked these with provided details of social positioning. To identify details which would provide an understanding of *power dynamics* we conducted a deductive thematic analysis using key aspects from the applied intersectional framework. To understand which aspects can be linked to *inequity and inequality* we searched primarily for details addressing the lived experiences of the person with dementia and/or their caregivers.

#### Axes of identity and social positions

Several axes of identity and social positioning were analyzed across the 13 included articles.

1. Migration status/ethnicity/race: these categories are understood as central to understanding stigma, barriers to access to care or the health care system and/or values of care (Ahmad et al., 2022; Altinok et al., 2025; Wong et al., 2024).
2. Socioeconomic position: this social position is mostly linked to immigration status, professional job and/or welfare system access and use, and analyzing how these intersections shape health care, e.g., what kind of care services are affordable and/or accessible (Ahmad et al., 2022; Liu et al., 2022; Roes et al., 2022).
3. Place and legal status: geographic location (e.g., urban vs. rural, country of residence) and legal status impact, for example eligibility, proximity to appropriate services and exposure to discrimination (Ahmad et al., 2022; Roes et al., 2022; Zacher et al., 2023).
4. Language and culture: these are intersecting identity markers linked to communication with the health care system as well as perceptions and concepts of dementia (Altinok et al., 2025; Wong et al., 2024).
5. Sex and gender: these are analyzed at the intersection of kin role within the care requirements and dynamics in care situations and highlight gendered expectations and burden (Liu et al., 2022; Misiura et al., 2023; Shippee et al., 2024). Sexual and gender minority groups with dementia experience discrimination at three different levels (individual, interpersonal, institutional) when living in a nursing home or in a heteronormative (exclusively) long-term care facility in which staff have various knowledge gaps (Shippee et al., 2024).

#### Analysis of power dynamics

The application of the intersectionality frameworks with a specific focus on power dynamics was not addressed directly in any of the 13 included articles.

- Ahmad et al. (2022) analyzed the effects of transnational migration processes, gender norms, and labor structures on who provides care and who receives care, applying Hankvisky's framework. They showed that migrant women are more often working as unpaid carers while navigating precarious employment and restrictive welfare services. The authors conclude that these results illustrate structural and institutional power within the healthcare system.
- Mwale et al. (2025) focus lies on institutional power at the intersection of age, race/ethnicity, socioeconomic status and dementia in acute hospitals. Applying the framework developed by Collins, they analyzed how these intersections impact hospital experience (e.g., if and how care needs are recognized). The authors conclude that privilege and marginalization are the product of the power dynamics between identity axes and institutional regulations and norms.
- Roes et al. (2022) describe how different intersectionality frameworks (Crenshaw, McCall, Hanskivsky) can be used to uncover discrimination, ignorance and exclusion of population groups (e.g., PLWD with a migration background) in dementia regulations or treatment and care strategies. The authors conclude that an intersectional analysis must extend beyond describing intersections toward interrogating structural power and policy-level neglect.
- Wong et al. (2024) apply an intra-categorial approach (McCall) to analyze how race (ethnicity), culture and immigration status interact with dementia and experiences of stigma, discrimination and social exclusion. The analysis revealed that power relations are visible in narratives of different treatments by healthcare professionals. The authors conclude that the individual experiences can be explained based on immigration-related power structures.

#### Understanding inequity and inequality

In some of the included articles we were able to see reasoning regarding experiences of inequity and inequality. Inequality refers to an unequal distribution of resources (e.g., differences in wealth), whereas inequity refers to unfairness and injustice (e.g., access to dementia services). Inequity is the result of human behavior, social structures and/or policies, and therefore can be avoided.

In the included articles four topics were addressed:

- PLWD experience different levels of access to diagnosis and dementia care services on the basis of a combination of intersections of migration status/ethnicity, language barriers, cultural expectations, and a low socioeconomic level. The interlockings of these intersections can be used to explain delayed diagnostic processes and challenges navigating the health care system as well as accessing/utilizing formal services (Ahmad et al., 2022; Altinok et al., 2025; Roes et al., 2022; Wong et al., 2024).
- Experiences of care burden intersect with sex/gender and migration status which is also interlocked with the unequal distribution of unpaid work and is constraint by (difficult) economic conditions, which reinforces inequalities (Ahmad et al., 2022).
- PLWD experience double discrimination due to the diagnosis and race/ethnicity/migration history. These experiences lead to social isolation and the misrecognition of dementia care needs. An analysis of these experiences through a lens of power dynamics and oppression shows that they are not just individual prejudices but structural manifestations of marginalization (Wong et al., 2024).
- PLWD experience institutional ignorance at the intersection of dementia diagnosis, age, ethnicity/race/migration status and socioeconomic status. In acute hospitals, they face mostly ignorant behaviors related to their needs, preferences, and voice. This can be understood as reproducing institutionalized inequities for PLWD who need acute care (Mwale et al., 2025).

## Discussion

With our scoping review, we show that although intersectionality frameworks have been applied in dementia care studies, theoretically grounded intersectional work remains sparse. Our analysis revealed a few research gaps: (a) underrepresentation of minority groups in dementia care research; (b) primarily focus lies on intersections, not on the analysis of underlying power dynamics; (c) unclear methodology how respective frameworks are applied; (d) theoretical background applied to analyze a phenomenon was in most cases not defined.

One of the greatest gaps lies in the *underrepresentation* of minority groups/ marginalized identities (Adames et al., 2020; Hafford-Letchfield, 2024; Wong et al., 2024) in research, resulting in a lack of tailored data. Another gap is that only a few studies have operationalized intersectionality with robust definitions and/or measures. Going back to the original framework of intersectionality (Crenshaw, 1989) and based on the included articles in this scoping review, it still seems necessary, to address the coloniality of power (Quijano, 2000) and its ongoing impact on knowledge production and language e.g., racial social classification of the world population, (Restrepo, 2018). For example, from the perspective of dementia research conducted in Europe we need to critically reflect on Eurocentric diagnostic norms (Nielsen, 2022), care models (Ahmad and The, 2025), research priorities and dementia strategies (Schmachtenberg et al., 2020). Due to the underrepresentation and missing critical reflection of production of knowledge and power, psychosocial interventions will not be tailored to the needs of care recipients with intersecting marginalized identities, and assessments lack validation for diverse populations.

Most studies mention the *intersection of multiple axes of identity*, but consider race/ethnicity/migration, sex/gender, age, or socioeconomic status more as separate covariates or descriptive subgroups. None of the studies have conducted intersectional power analyses or used one of the most cited intersectionality frameworks to directly analyze power dynamics to understand reasons for experiences of inequity and/or inequality. For example, power dynamics that operate through institutional *cultures of control* (Voruz, 2005) and/or everyday rules. Analyzing power dynamics requires more than simply documenting unequal outcomes. Thus, the gap lies between deciding to use an intersectionality framework and its operationalization as a critical analysis of the interlocking systems and axes of identity in dementia care research designs, analysis, and interpretations.

From a conceptual perspective, almost all included articles considered a combination of at least two intersectionality frameworks. Unfortunately, *methodological details* on how the frameworks were applied, *were missin*g. From our perspective, the provision of methodological details on how to apply the intersectionality framework (e.g., IBPA, McCall's categorial system, or Bowleg's health equity guidance) would support strong performances and helps identify asymmetries within the various health care systems; for example combining intra-categorical qualitative approaches (for deep analysis of marginalized intersections) with inter-categorical perspectives (to compare patterns across intersections), and explicitly locating micro-experiences within structural determinants. An exception is the study from Mwale et al. (2025) on theorizing intersectionality and cultures of control of people living with dementia in acute hospital settings.

Moreover, dementia care research needs to be expanded by using relevant *conceptual models and theoretical perspectives*, for example, gender theory and/or critical feminist-post-humanist approaches (Finn and Brown, 2022). Since most family caregivers are women, it is no surprise that intersectionality and feminist / gender theories are emerging in dementia studies. For example, Bartlett (2022) criticizes that sex/gender neutrality (e.g., not including the sex of the caregiver) appears as a negative way of diminishing sex/gender in dementia care studies. She points out, that gender neutral language (people with dementia) can be viewed as a way of excluding this aspect of the identity in dementia care setting. However, a gender-neutral language could be beneficial for people who do not agree with existing sex and gender norms (Lotherington, 2023). For example, sexual and gender minoritized (SGM) people living with dementia have explicitly addressed ways in which multiple forms of oppression (e.g., ethnocentrism, cis-sexism, heterosexism) uniquely impact their experiences of inequity and/or inequality (Adames et al., 2020; Europe, 2021; Stites and Velocci, 2024).

Furthermore, there is a need to advance dementia care research. Moving away from treating demographic variables as independent confounders toward understanding the intersections, the structural systems of power, which lead to inequities and inequalities. We recommend three key actions.

1. Methodological and analytical advancements: there is a need to move beyond simple comparison (e.g., white vs. minority groups) and on to analyze the combined impact of the overlapping intersections (e.g., living in a rural and poor neighborhood with a biography of being a migrant) on patient related outcomes (Tobin Thomas et al., 2023). Conducting multilevel analyses of individual heterogeneity and discriminatory accuracy (MAHIDA) is a quantitative, intersectional approach to analyze socio-demographic inequalities (Wilkes and Karimi, 2024). Applying mixed-method approaches to analyze the how and why of intersecting experiences and striving for global equity data science (Vilor-Tejedor et al., 2026) is needed too.
2. Integrating health disparities and structural factors: there is a need to analyze how structural determinants impact disparities in dementia incidence/prevalence, diagnosis, treatment and care (Balls-Berry and Babulal, 2022). The analysis of double jeopardy of individual holding multiple marginalized identities (e.g., people with intellectual and developmental disabilities) will help to explain patient reported outcomes (Marks et al., 2025).
3. Reconsider research designs: there is a need to reconsider recruitment strategies specifically from populations facing high level of inequity (e.g., LGBTQI+, ethnic minority groups, indigenous communities) to ensure adequate sample size for intersectional analysis (Maestre et al., 2024). The implementation of community-based-participatory research seems to be an effective way to ensure that research topics are relevant to marginalized communities (Collins et al., 2018), which also has an impact on implementing innovative interventions (Fischer et al., 2025). Additionally, developing longitudinal datasets might lead to a better understanding of structural determinants (e.g., neighborhood deprivation) and its impact on patient reported outcomes (Vilor-Tejedor et al., 2026).

## Conclusion

Our scoping review is to our knowledge the first attempt to critically analyze the application of intersectionality frameworks in dementia care research. The development of intersectional frameworks began with the aim of conceptually advocating for population groups (such as Black women) that are underrepresented (ignored or defined as hard to reach) and has progressed to offering a variation of different frameworks as well as methods and research applications. There are some indications, that intersectionality frameworks are now used as both theoretical and analytical strategies by specifying axes of identity and the location of power dynamics within systems of power, and furthermore in analyzing the narrative experiences, to better understand inequity and inequality.

Among the included articles, most focused on analyzing the intersection of axes of identity, but these analytical results were not viewed through the lens of power dynamics and oppression in all cases. Based on the similarities of all intersectionality frameworks, solely analyzing the intersection of identity axes is insufficient to understand experiences of inequity and inequality. An intersectional power analysis would need the following: (a) an explicitly defined system (e.g., sexism, migration regulations) that interlocks with observed outcomes of oppression; (b) a connection between individual experiences (e.g., stigma, exclusion) and different levels of organizational/institutional norms (e.g., admission practices to acute hospitals or access to care services) and policies (e.g., national dementia plans or professional guidelines); and (c) an understanding of the axes of identity and power dynamics within a society (or the health care system) as historically situated and relational (i.e., interlocking).

We believe that the recent increase in the adoption of intersectional approaches will enable dementia care research that analyzes not only different categories or social axes of identity but also the dynamics and relations between these categories embedded in their specific context and how they are produced by their conjunction (Dilworth-Anderson et al., 2020).

## Limitations

We primarily focused on studies and articles from the (dementia) health care research spectrum. We therefore may have missed other intersectionality frameworks applied in other disciplines, such as anthropology. For example, sociocultural anthropologists value origin stories and understand them as windows into social worlds as well as treasure origin stories to recognize the history of a story (Miller, 2025; Thimm, 2025).
