# Supplementary material for: Mapping intersectionality in dementia care research: a decadal scoping review
Source: Front Dement. 2026 Sep 3;5:1779670. doi: 10.3389/frdem.2026.1779670 (PMC13581494; doi:10.3389/frdem.2026.1779670)
Supplement: Supplementary file 1 [file Supplementary_file_1.docx]

1 **Supplement** (different search strings)

**PubMed**

((dementia[Title/Abstract] OR Alzheimer*[Title/Abstract])) AND (intersectionality[Title/Abstract] OR intersectional[Title/Abstract] OR "multidimensional identity"[Title/Abstract] OR "structural inequality"[Title/Abstract] OR disparities[Title/Abstract] OR migrants[Title/Abstract] OR "minority group"[Title/Abstract] OR "social determinants"[Title/Abstract]) AND (care[Title/Abstract] OR caregivers[Title/Abstract] OR healthcare[Title/Abstract] OR support[Title/Abstract] OR service[Title/Abstract]) AND (framework[Title/Abstract] OR analysis[Title/Abstract] OR approach[Title/Abstract] OR model[Title/Abstract])

**PsychINFO**

(AB(dementia OR Alzheimer*)) AND AB(intersectionality OR intersectional OR "multidimensional identity" OR "structural inequality" OR disparities OR migrants OR "minority group" OR "social determinants") AND AB(care OR caregivers OR healthcare OR support OR service) AND AB(framework OR analysis OR approach OR model)

**Scopus**

(TITLE-ABS-KEY(dementia OR Alzheimer*) AND TITLE-ABS-KEY(intersectionality OR intersectional OR "multidimensional identity" OR "structural inequality" OR disparities OR migrants OR "minority group" OR "social determinants") AND TITLE-ABS-KEY(care OR caregivers OR healthcare OR support OR service) AND TITLE-ABS-KEY(framework OR analysis OR approach OR model))

**CINAHL**

(dementia OR Alzheimer*) AND (intersectionality OR intersectional OR "multidimensional identity" OR "structural inequality" OR disparities OR migrants OR "minority group" OR "social determinants") AND (care OR caregivers OR healthcare OR support OR service) AND (framework OR analysis OR approach OR model) [Search in “Abstract” or “Title” fields if supported]

**Embase**

('dementia':ab,ti OR 'Alzheimer*':ab,ti) AND ('intersectionality':ab,ti OR 'intersectional':ab,ti OR 'multidimensional identity':ab,ti OR 'structural inequality':ab,ti OR 'disparities':ab,ti OR 'migrants':ab,ti OR 'minority group':ab,ti OR 'social determinants':ab,ti) AND ('care':ab,ti OR 'caregivers':ab,ti OR 'healthcare':ab,ti OR 'support':ab,ti OR 'service':ab,ti) AND ('framework':ab,ti OR 'analysis':ab,ti OR 'approach':ab,ti OR 'model':ab,ti)

**Web of Science**

TS=(dementia OR Alzheimer*) AND TS=(intersectionality OR intersectional OR "multidimensional identity" OR "structural inequality" OR disparities OR migrants OR "minority group" OR "social determinants") AND TS=(care OR caregivers OR healthcare OR support OR service) AND TS=(framework OR analysis OR approach OR model)

Table 2: Study characteristics (alphabetical order)

| **Authors** | **Title** | **Intersectionality Framework** | **Research Question / Aim** | **Target Group** | **Key Outcomes** |
| --- | --- | --- | --- | --- | --- |
| Ahmad et al (2022) | Dementia care-sharing and migration: An intersectional analysis of family care practices | Crenshaw  Hankivsky | To explore how intersecting social categories shape care-sharing practices and responsibilities in families affected by dementia in a migration context. | Migrant families providing care to relatives with dementia (e.g., adult children, spouses, extended kin). | - Highlights unequal care burdens for migrant women and how structural conditions (labor markets, welfare rules) shape intrafamiliar negotiations - how gender, migration trajectories, class and legal status interact to distribute and constrain care roles |
| Altinok et al (2025) | Intersectional research on dementia care for post-migrants and ethnic minority groups: a scoping review | McCall, Crenshaw | To identify the range, extent and nature of evidence available in peer-reviewed and gray literature and to examine how the intersecting experiences and differences of post-migrants and ethnic minority groups influence preferences. | Post-migrants & ethnic minorities people with dementia and carers | - Preferences are not linked to intersectional determinants - Personal preferences are shaped by intersecting cultural values, language barriers, immigration processes, shifting socioeconomic positions and gender roles - Underscores system-level neglect of these intersections in service planning. |
| Azizan et al (2025) | A research landscape analysis on Alzheimer's disease and gero-technology: Identifying key contributors, hotspots, and emerging trends | Collins,  McCall | What intersections define research gaps in Alzheimer’s care? | Alzheimer’s Dementia populations | - Technology/care needs vary by SDH intersections - Integrate intersectionality in research design |
| Bartlett et al (2018); Bartlett & O'Connor (2010) | Gender, citizenship and dementia care: a scoping review of studies to inform policy and future research | Crenshaw,  Gender theory | To take stock of knowledge about gender differences in relation to dementia care to inform policy and future research | People with dementia  living in the  U.K. or USA | - Need to address gender, migration, policy together - Intersectionality essential for inclusion |
| Bartlett (2022) | Inclusive (social) citizenship and persons with dementia | Crenshaw,  Gender theory | How does intersectionality redefine selfhood in care? | People with dementia and caregivers | - Identity and agency reconstructed across intersecting axes - Agency depends on intersectional recognition |
| Ghanem et al (2025) | Key considerations for applying intersectionality theory to partner and stakeholder engagement in public health | Hanckivsky | How is intersectionality defined and operationalized in health research literature? | Different stakeholders | - Calls for incorporating intersectionality principles in partner-stakeholder engagement (PSE) in public health - Encouraging critical reflection on the who, why, and how of PSE |
| Liu et al (2022) | Caregiving Burden Among Caregivers Through Lens of Intersectionality | Crenshaw, McCall,  Bowleg | How do race/gender intersections shape burden among caregivers? | Informal caregivers | - Ethnicity/gender intersections yield distinct burdens - Tailored interventions are needed |
| Misiura et al (2023) | Intersectionality in Alzheimer’s Disease: The Role of Female Sex and Black American Race in the Development and Prevalence of AD | McCall | How do female sex and Black American race intersect to affect Alzheimer’s Disease risk, prevalence, and outcomes? | Black female Americans with Alzheimer’s Disease | - Social and biological factors, such as allostatic load and hormone use, intersect to increase Alzheimer’s Disease risk |
| Mwale et al (2025) | Privileges, and Permissions: Theorising Intersectionality and Cultures of Control in the Care of People Living With Dementia in Acute Hospital Settings | Collins | How to conceptualize privilege and permission are distributed in acute dementia care, linking identity axes to institutional power | Dementia patients in acute hospitals | - Institutional power systems drive inequalities and intersectionality exposes structures - Critique and deconstruct frameworks of exclusion and control embedded in hospital cultures |
| Roes et al (2022) | Intersectionality and its relevance for research in dementia care of people with a migration background | Crenshaw, McCall, Hanckivsky | How does intersectionality illuminate exclusion for migrants with dementia? | Migrants with dementia | - Interlocking identities reveal systemic care barriers - Advancing intersectionality needed to understand exclusions from access to care |
| Sherman et al (2023) | Intersectionality in nursing research: A scoping review | Crenshaw, Collins | How is intersectionality used in nursing? | Nursing/dementia care recipients | - More explicit use of intersectionality frameworks needed |
| Shippee et al (2024) | Scoping Literature Review: Experiences of Sexual and Gender Minority Older Adults With Diagnoses of Dementia, Who Use Residential Long-Term Services and Supports | Crenshaw | What are the experiences and challenges faced by SGM older adults with dementia in residential LTSS? | Sexual & gender minority Older adults with dementia | - Sexual, gender, minority (SGM) older adults encounter compounded discrimination - Risk is magnified (e.g., transgender, racial minorities, HIV+) - Facility and staff often remain inadequate |
| Zacher et al (2023) | Geographic Patterns of Dementia in the United States: Variation by Place of Residence, Place of Birth, and Subpopulation | McCall | Evaluate, whether and how risk for assessed dementia varies by place of residence and birth overall and by race/ethnicity and education. | People with dementia living in the US | - Disadvantage and nuanced patterns of dementia prevalence embedded within place and subpopulation groupings |
| Wong et al (2024) | Living with dementia: Exploring the intersections of culture, race, and dementia, stigma | McCall | To explore how culture, race and immigration shape experiences of living with dementia and  dementia-related stigma. | Immigrant PWLD and caregivers living in Canada | - Identifies how racism, cultural norms, immigration histories and cognitive impairment interact - How they produce layered stigma, social exclusion, and barriers to support - Highlights power in health encounters and service access. |
