# Supplementary material for: Mapping intersectionality in dementia care research: a decadal scoping review
Source: Front Dement. 2026 Sep 3;5:1779670. doi: 10.3389/frdem.2026.1779670 (PMC13581494; doi:10.3389/frdem.2026.1779670)
Supplement: Supplementary file 2 [file Supplementary_file_2.docx]

Table 3: Study characteristics (alphabetical order)

| **Authors** | **Title of the article** | **Intersectionality Framework** | **Research Design & methods** | **Population** | **Key Findings** |
| --- | --- | --- | --- | --- | --- |
| Ahmad et al (2022) | Dementia care-sharing and migration: An intersectional analysis of family care practices | **Crenshaw** (1989, 1991) [original intersectionality concept] and **Prins** (2006) [a systemic approach foregrounding structure and a constructionist approach foregrounding agency] and **Phoenix** (2006, 2011) [that researchers should inductively decide which intersections matter in context] | **Qualitative**:  Life-story interviews and shadowing | Migrant family carers providing care to relatives with dementia | - Highlights unequal care burdens for migrant women and how structural conditions (labor markets, welfare rules) shape intra-familiar negotiations - how gender, migration trajectories, class and legal status interact to distribute and constrain care roles |
| Altinok et al (2025) | Intersectional research on dementia care for post-migrants and ethnic minority groups: a scoping review | **Crenshaw** (1989, 1991)  [original intersectionality concept] | Scoping **review** | Post-migrants & ethnic minorities people with dementia and carers | - Preferences are not linked to intersectional determinants - Personal preferences are shaped by intersecting cultural values, language barriers, immigration processes, shifting socioeconomic positions and gender roles - Underscores system-level neglect of these intersections in service planning. |
| Bartlett et al., 2018 | Gender, citizenship and dementia care: a scoping review of studies to inform policy and future research | Single-axis gender lens  (**Butler** 1990; **Connell** 1987 **West & Zimmermann** 1987) to expose gendered norms, policy discourses and care practices | Scoping **review** | Family carers of people with dementia | - Need to address gender, migration, policy together - Intersectionality essential for inclusion |
| Ghanem et al. (2025) | Key considerations for applying intersectionality theory to partner and stakeholder engagement in public health | **Hanckivsky** (2012) [Intersectionality-Based Policy Analysis (IBPA) Framework] to provide a method on how to analyse health policy (reports) | Literature **review** | Different stakeholders | - Calls for incorporating intersectionality principles in partner-stakeholder engagement (PSE) in public health - Encouraging critical reflection on the who, why, and how of PSE |
| Mwale et al (2025) | Privileges, and permissions: theorising intersectionality and cultures of control in the care of people living with dementia in acute hospital settings | **Collins & Bilge** (2016, 2020) concept of intersectionality and relational power | **Ethnographic** observations | Dementia patients in acute hospitals in UK | - Institutional power systems drive inequalities and intersectionality exposes structures - Critique and deconstruct frameworks of exclusion and control embedded in hospital cultures |
| Liu et al (2022) | Caregiving Burden Among Caregivers Through Lens of Intersectionality | **Crenshaw** (1989) [original intersectionality concept] and **Hankivsky** (2012) [health equity intersectionality framework] an **McCall** (2005) [methodology] | **Quantitative**:  Logistic regression | Informal caregivers | - Ethnicity/gender intersections yield distinct burdens - Tailored interventions are needed |
| Roes et al. (2022) | Intersectionality and its relevance for research in dementia care of people with a migration background | **Bowleg**  **Collins** (2019) [intersectionality concept]; **Crenshaw** (1989) [original intersectionality concept]; **Hanckivsky** (2012) [Intersectionality-Based Policy Analysis (IBPA); **McCall** (2005) three different categorical approaches | Perspective Paper | People with dementia and migration history | - Interlocking identities reveal systemic care barriers - Advancing intersectionality is needed to understand exclusion from dementia care |
| Sherman et al. (2023) | Intersectionality in nursing research: A scoping review | Provides a brief overview of **different intersectionality frameworks** (e.g. Crenshaw, Bowleg),  not dementia research specific | Scoping **review** | Nursing/dementia care recipients | - More explicit use of intersectionality frameworks needed |
| Wong et al (2024) | Living with dementia: Exploring the intersections of culture, race, and dementia, stigma | **Crenshaw** (1989) [original intersectionality concept] and **Collins** (2019) [intersectionality as a critical social theory] and Applying **McCall’s** (2005) intra-categorical approach | **Qualitative**:  interviews | Immigrant PWLD and caregivers living in Canada | - Identifies how racism, cultural norms, immigration histories and cognitive impairment interact - Producing layered stigma, social exclusion, and barriers to support - Highlights power in health encounters and service access. |
| **Authors** | **Title of the article** | **Focus on intersections,**  **not explicit an  intersectional framework** | **Research Design & methods** | **Population** | - **Key Findings** |
| Bartlett (2022) | Inclusive (social) citizenship and persons with dementia | **Single-axis** gender lens  to argue for includes social citizenship | **Qualitative**:  Go-along walking followed by sit-down interviews | People with dementia and caregivers in England | - Identity and agency reconstructed across intersecting axes - Agency depends on intersectional recognition |
| Misiura et al (2023) | Intersectionality in Alzheimer’s Disease: the role of female sex and Black American race in the development and prevalence of AD | **intersecting axes** of sex and race, such as female biological sex, Black American race/ethno‑racial identity, and a set of social determinants of health (poverty, education, discrimination, allostatic load, vascular risk factors, access to care, under‑representation in trials) | Selective literature **review** | Black female Americans with Alzheimer’s Disease | - Social and biological factors, such as allostatic load and hormone use, intersect to increase Alzheimer’s Disease risk |
| Shippee et al (2024) | Scoping literature review: experiences of sexual and gender minority older adults with diagnoses of dementia, who use residential long-term services and supports | **McLeroy**, (1988) [Socio-ecological model] focus on how individual, interpersonal, organizational, community and policy‑level factors **interact** to shape health outcomes | Scoping **review** | Sexual & gender minority older adults with dementia | - Sexual, gender, minority (SGM) older adults encounter compounded discrimination - Risk is magnified (e.g., transgender, racial minorities, HIV+) - Facility and staff often remain inadequate |
| Zacher et al (2023) | Geographic Patterns of Dementia in the United States: Variation by Place of Residence, Place of Birth, and Subpopulation | **Intersection** of place and subpopulation using a life course and social special health disparity lens (e.g. Akushevich et al., 2021)) | **Quantitative**: Logistic regression | People with dementia  living in the US | - Disadvantage and nuanced patterns of dementia prevalence embedded within place and subpopulation groupings |
