# Supplementary material for: Mapping intersectionality in dementia care research: a decadal scoping review
Source: Front Dement. 2026 Sep 3;5:1779670. doi: 10.3389/frdem.2026.1779670 (PMC13581494; doi:10.3389/frdem.2026.1779670)
Supplement: Supplementary file 3 [file Supplementary_file_3.docx]

**Identification of articles via databases**

Articles removed *before screening*:

duplicates removed
(n = 320)

Articles identified from different databases (PubMed, PsycINFO, Scopus, CINAHL, Embase, Web of Science)

(n = 870)

**Identification**

Articles excluded (n = 440)

Gray literature

Conceptual/theoretical papers

Biomedical article

Articles screened

(n = 550)

Articles not retrieved

(n = 20)

Abstract only

No access to the article

Articles sought for retrieval

(n = 130)

**Screening**

Articles assessed for eligibility

(n = 110)

Articles excluded: 97

Reason 1- Focus on staff

Reason 2 – People with dementia only subgroup

Reason 3 – focus on technology

Articles included in review

(n = 13)

**Included**

Figure 1: PRISMA Flowchart
